# Incidence, classification, healing patterns, and vascular remodeling of radial artery dissection assessed by OCT

**DOI:** 10.3389/fcvm.2026.1754057

**Published:** 2026-06-03

**Authors:** Hao Liu, Jia Zhou, Saiying He, Senhu Wang, Haotian Wang, Zixuan Li, Rui Yan, Jincheng Guo

**Affiliations:** 1 Department of Cardiology, Beijing Luhe Hospital, Capital Medical University, Beijing, China; 2 Emergency Department, Beijing Luhe Hospital, Capital Medical University, Beijing, China

**Keywords:** dissection, optical coherence tomography, percutaneous coronary intervention, radial artery, remodeling

## Abstract

**Background:**

The incidence and natural history of radial artery dissection (RAD) after transradial or distal transradial coronary intervention (TDRI) remain unclear.

**Aims:**

To determine the incidence, classification, healing patterns, vascular remodeling, and factors associated with RAD identified by optical coherence tomography (OCT).

**Methods:**

This retrospective single-center study included 1,995 patients who underwent radial artery OCT during TDRI with a uniform 6 Fr introducer sheath between 2016 and 2024. RAD incidence was assessed on a patient basis (≥1 RAD/patient). RAD lesions were classified per lesion as Type I (flap) or Type II (cavity), stratified by external elastic membrane (EEM) depth (E1 within media; E2 at EEM; E3 beyond EEM). Healing and remodeling were analyzed per lesion in a repeat-OCT cohort defined as patients who underwent repeat radial artery OCT during subsequent TDRI; a remodeling index <1 defined negative remodeling. Exploratory logistic regression was performed to identify factors associated with RAD.

**Results:**

RAD occurred in 266/1,995 patients (13.3%). Overall, 325 RAD lesions were identified (mean 1.22 per patient); the most frequent baseline subtype was Type I–E1 (33.8%). In exploratory multivariable analysis, older age, female sex, longer procedure duration, clinically relevant radial artery spasm, and smaller OCT-derived reference radial artery diameter remained associated with RAD. In the repeat-OCT cohort (22 patients; 28 lesions), repeat OCT at day 9 in one patient (3 lesions) showed partial healing, whereas repeat radial artery OCT performed ≥30 days in the remaining 21 patients (25 lesions) demonstrated complete healing, predominantly through intimal hyperplasia (60%) or fibrous plaque (40%). The mean remodeling index was 0.94 ± 0.11.

**Conclusions:**

In this 6 Fr sheath-based TDRI cohort,RAD was frequently detected by OCT and was usually limited in depth. Older age, female sex, radial artery spasm, longer procedure duration, and smaller radial artery diameter were associated with RAD. lesions reassessed ≥30 days healed completely with mild negative remodeling.

## Graphical Abstract

**Figure:**
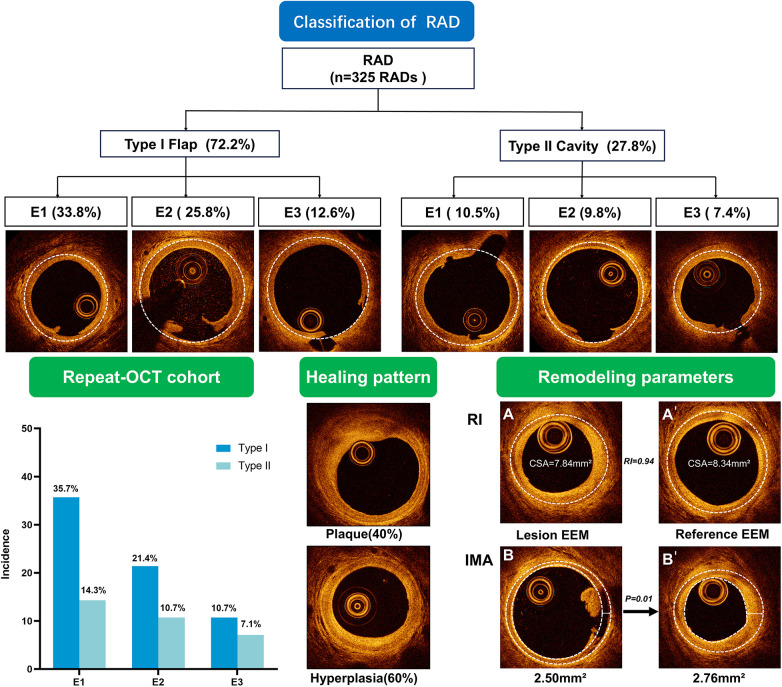
The upper panel summarizes the OCT-based classification of 325 RAD lesions into Type I flap and Type II cavity dissections, with E1–E3 indicating increasing depth of vessel wall injury relative to the external elastic membrane. The lower panels summarize findings in the repeat-OCT cohort of 22 patients with 28 lesions, including subtype distribution, healing patterns, and remodeling assessment by RI, lesion/reference EEM area, and intima–media area. EEM, external elastic membrane; IMA, intima–media area; OCT, optical coherence tomography; RAD, radial artery dissection; RI, remodeling index.

## 1 Introduction

Transradial or distal transradial access has become the preferred approach for percutaneous coronary interventions (PCI) (1). However, transradial or distal transradial interventions (TDRI) can be associated with various complications, among which radial artery dissection (RAD), with or without perforation, represents a potentially serious vascular injury. RAD typically results from improper or forceful manipulation of the introducer sheath or guiding catheter, leading to structural damage to the arterial wall (2). Although previous studies suggest that RAD can often be sealed spontaneously by the catheter itself (3), vessel wall injury may provoke endothelial dysfunction, thrombogenic activation, and local inflammation, which in turn can contribute to intimal hyperplasia, luminal narrowing, or even long-term occlusion (4), These adverse vascular responses may ultimately compromise the future usability of the radial artery for repeat TDRI or as a conduit in coronary artery bypass grafting.

Conventional imaging modalities, such as radial artery angiography (5) and ultrasound (6) have been used to detect RAD, with reported incidence rates ranging from 1.2% to 10%. However, optical coherence tomography (OCT), with its superior axial resolution, has revealed markedly higher rates of acute RAD, ranging from 16.5% to 35.6% (7, 8). These findings, derived from small observational cohorts, provide limited insight into the true prevalence.

Current understanding of the natural history of vascular dissections is largely derived from studies on coronary stent edge dissection (SED), which have demonstrated a high rate of spontaneous healing within 12 months, as evidenced by serial OCT imaging (9, 10). In contrast, limited data exist regarding the temporal progression, healing mechanisms, and clinical relevance of RAD.

Accordingly, this study sought to: (1) quantify the patient-level incidence of OCT-defined RAD after TDRI and classify RAD morphology on a per-lesion basis; and (2) in a repeat-OCT, characterize per-lesion healing patterns and evaluate vascular remodeling using the remodeling index.

## 2 Methods

### 2.1 Study design and patient selection

**Figure 1:**
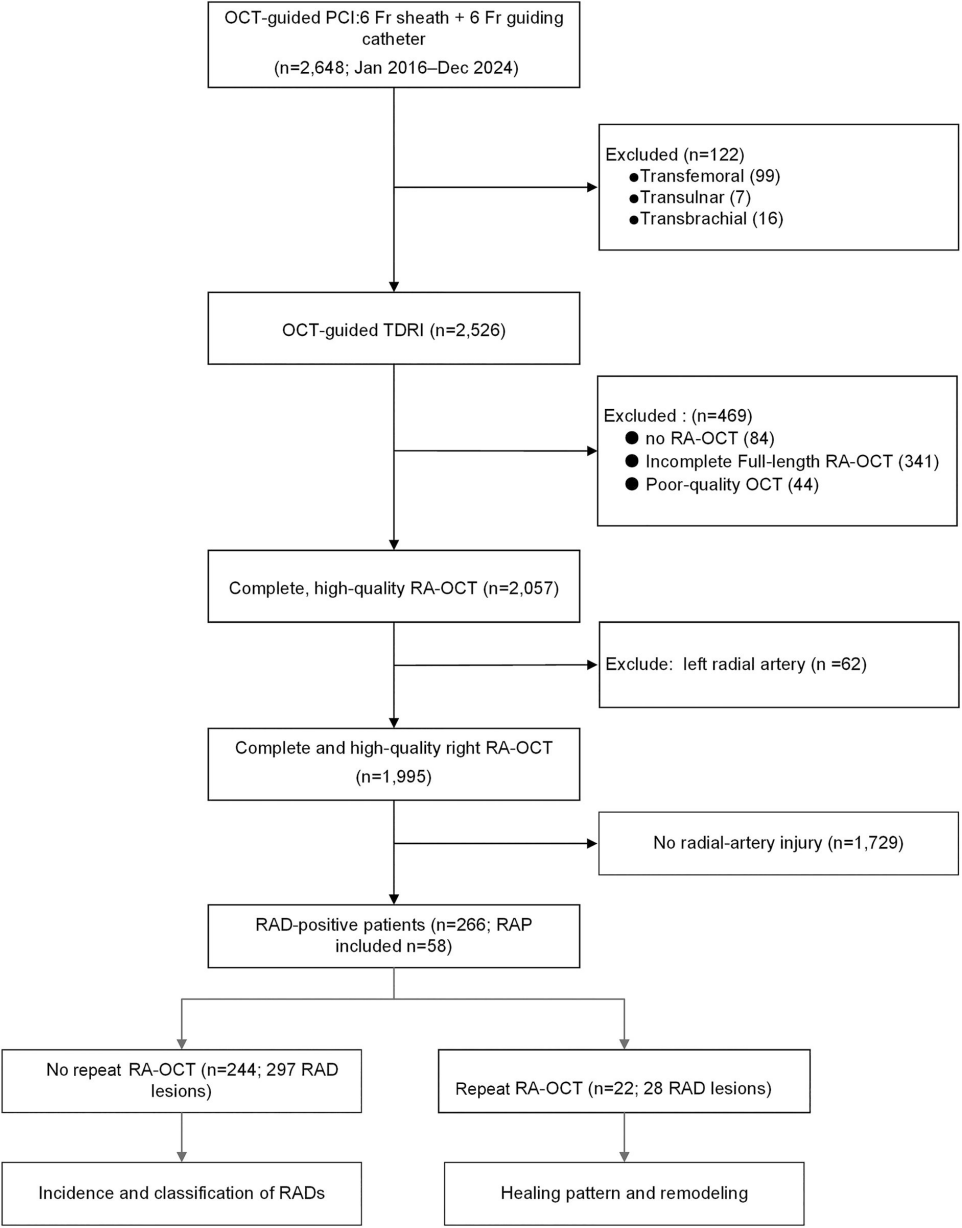
Study flowchart. Among 2,526 patients undergoing OCT-guided TDRI (2016–2024), 2,057 had analyzable radial-artery OCT (RA-OCT). After excluding left-radial access (*n* = 62), 1,995 right-radial patients were evaluated for RAD incidence. OCT, optical coherence tomography; PCI, percutaneous coronary intervention; RA-OCT, radial artery optical coherence tomography; RAD, radial artery dissection; RAP, radial artery perforation; TDRI, transradial or distal transradial coronary intervention.

This single-center, retrospective observational study included consecutive patients who underwent OCT-guided TDRI with subsequent full-length OCT of the right radial artery (from the ostium to 2 cm proximal to the puncture site) at Beijing Luhe Hospital between January 2016 and December 2024. Patients with analyzable full-length radial OCT pullbacks were entered into the incidence cohort for assessment of RAD occurrence. Among patients with OCT-confirmed RAD at baseline, those who underwent repeat full-length radial OCT via the same vascular access during a later procedure comprised the repeat-OCT cohort, in which RAD morphology, healing patterns, and vascular remodeling (by remodeling index) were evaluated. The study flow is summarized in Figure 1. Exclusion criteria were: (1) poor-quality or incomplete OCT imaging, or (2) declined participation. This study was approved by the Institutional Review Board of Beijing Luhe Hospital, Capital Medical University (Approval No. 2024-LHKY2025-02). Informed consent was obtained from all participants. The study protocol is registered at chictr.org under the title “Mapping the Distribution of Radial Artery Diameter, Atherosclerosis, Acute and Chronic Injury by Optical Coherence Tomography” (ChiCTR2500097574).

### 2.2 PCI and OCT examination procedure

The right radial artery or distal radial artery was selected as the access route for TDRI. After successful puncture, a 0.025-inch guidewire was advanced, followed by insertion of a 6F, 16-cm Radiofocus Introducer II sheath (Terumo Corp., Tokyo, Japan).,which was used in all procedures. Coronary angiography was generally performed using a 5 Fr multipurpose diagnostic catheter, followed by PCI with a 6 Fr guiding catheter. In selected patients, particularly those with ST-segment elevation myocardial infarction, coronary angiography and PCI were completed using a single 6 Fr guiding catheter to reduce catheter exchange, as previously described (11, 12)

After completion of coronary intervention, the guidewire was left in the brachial artery, and RA angiography was performed to identify the radial artery ostium. An external radiopaque ruler was aligned with the ostium level, and the sheath was retracted to a point 2 cm proximal to the puncture site. A vasodilator cocktail containing 200 µg of nitroglycerin and 2.5 mg of verapamil was administered via the sheath.

The OCT catheter was then advanced to the radial artery ostium over the guidewire. Before March 2019, OCT pullbacks were performed over the guidewire; from March 2019 onward, the guidewire was routinely withdrawn before pullback to avoid guidewire shadow without compromising image quality. Because the maximum pullback length was 54 mm for C7XR and 75 mm for OPTIS, complete radial artery imaging was performed using sequential pullbacks with stepwise distal catheter withdrawal. A representative off-wire OCT pullback with co-registration is provided in Supplementary Video S1. To clear blood from the imaging field, 2–3 mL of normal saline was manually injected through the catheter. Additional saline was infused via the sheath side port to flush the radial artery and facilitate image acquisition. Clinically relevant radial artery spasm was defined by operator documentation of forearm pain and/or resistance during radial guidewire, catheter, or sheath manipulation, supported by angiography when available (2).

Manual injection was synchronised with automated pullback at 20 mm/s. Each pullback covered either 54 mm (C7XR FD-OCT system, Abbott Vascular) or 75 mm (OPTIS Imaging System, Abbott Vascular, Santa Clara, CA, USA). Three to four pullbacks were performed to visualise the entire segment from the RA ostium to the tip of the sheath. Final RA angiography was performed at the end of the procedure. All procedures were conducted by three experienced interventional cardiologists, each with >10 years of experience and an annual volume of >200 cases.

### 2.3 OCT image analysis and definitions

Two trained physicians independently analyzed RAD using offline OCT image analysis software (OPTIS Imaging System, Abbott Vascular, Santa Clara, CA, USA). In cases of discrepancy, a third OCT expert adjudicated the results.

On OCT, RAD was defined as an intimal tear with disruption of the medial layer of the radial artery wall. The location of each RAD was determined as the distance from the RA ostium to the proximal edge of the dissection. RAD length was calculated by multiplying the number of consecutive OCT frames showing dissection by the axial frame interval (pullback speed [mm/s] ×frame rate [s]) (13).

RAD lesions were first classified into two morphological types based on their appearance on OCT. Type I (flap-type) dissections were defined as disruptions of the luminal vessel surface with one or more intima-media flaps protruding into the lumen. Type II, or cavity-type dissection, was defined as disruption of the luminal vessel surface with an underlying intramural cavity (14, 15). Each type was further sub-classified based on the depth of media involvement relative to the external elastic membrane (EEM): E0 (no dissection), E1 (within media), E2 (at EEM), and E3 (beyond EEM), each with or without hematoma (Graphical abstract). Hematoma was defined as the accumulation of blood within the media, separating the intima–media complex from the adventitia, with communication to the lumen (16). RAD with perforation was defined as complete loss of vessel wall integrity, analogous to type E3 (17).

**Figure 2:**
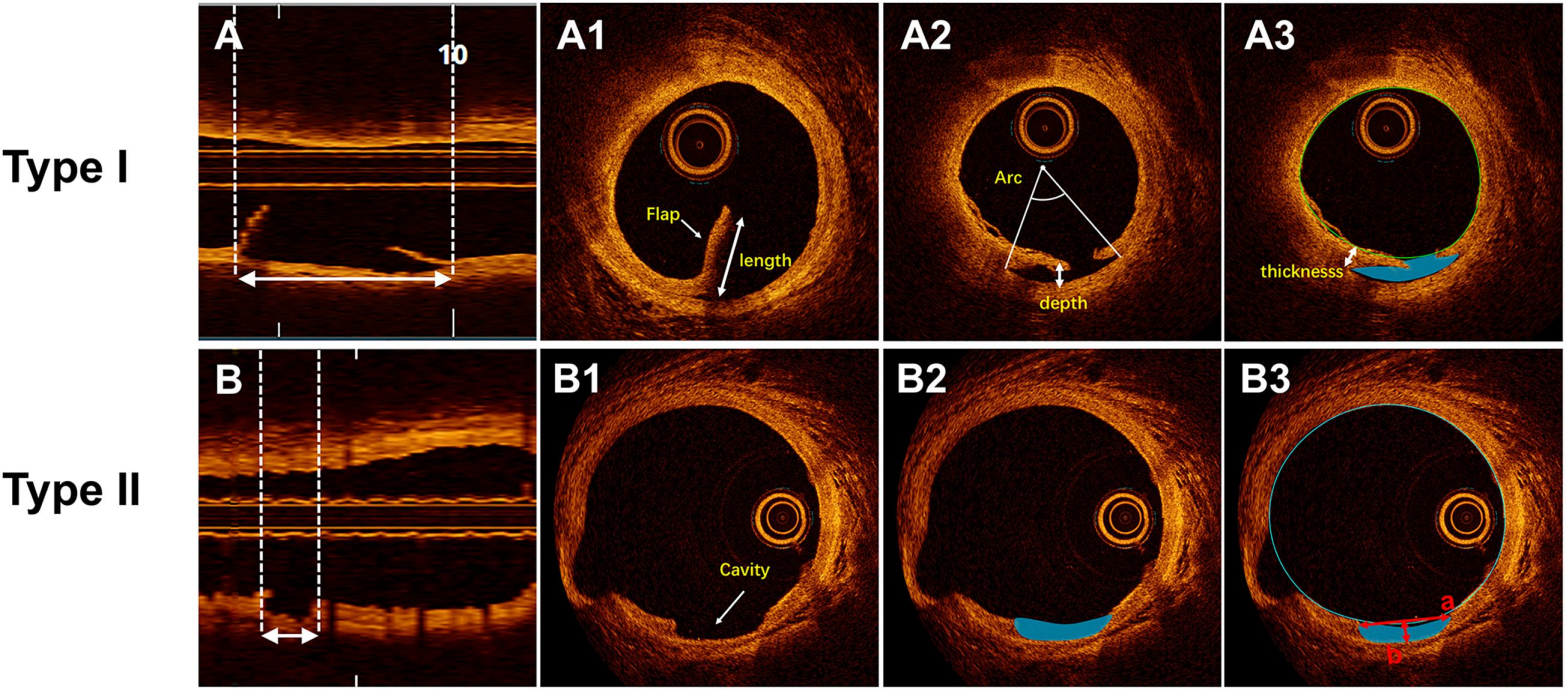
OCT-Based morphometric features and classification of radial artery dissection. **(A)** Longitudinal OCT views of Type I dissections characterized by one or two intima–media flaps, with dissection length indicated by a double arrowhead. Panels A1–A3 show flap length, arc, depth, and thickness.**(B)** Longitudinal OCT views of Type II dissections featuring an isolated intramural cavity, with dissection length indicated by a double arrowhead. Panels B1–B3 show the cavity (arrow), false lumen (blue shading), and cavity depth **(b)** and width **(a).**

The length of the free intimal flap was measured as the distance from the flap tip to its base, and its thickness was quantified at the base. Cavity area was planimetrically calculated as the area enclosed between the inner and outer luminal contours. The maximum inter-edge distance across the dissected segment was defined as cavity width. Cavity depth was defined as the perpendicular distance from the deepest point of the cavity to a virtual baseline connecting the luminal contours on both sides, measured along a line passing through the centroid of the lumen. The arc of dissection was measured from the vessel centre to determine its circumferential extent and was categorized as small (≤90°) or large (>90°) based on cross-sectional OCT images. A schematic overview of all measurements is shown in Figure 2.

To ensure consistent evaluation of arterial segments across baseline and repeat-OCT paired images were reviewed side by side for serial analysis. The assessment was guided by motorized pullback speed and identifiable anatomical landmarks such as calcified plaques, side branches, and overall plaque morphology. Corresponding frames across sequential OCT pullbacks were manually aligned using these landmarks (18).

Healing patterns of RAD were categorized as follows: Intimal hyperplasia was defined as as increased intimal thickness with preservation of the normal three-layer vessel wall architecture (19). Atherosclerotic plaques were identified by disruption or loss of the normal layered wall structure on OCT. Plaques were classified as fibrotic, Calcified, or lipid-rich according to previously described criteria (20). Vascular remodeling was assessed using the remodeling index, area stenosis, and intima–media area. Remodeling index $=\frac{EEM\;lesion\;area}{EEM\;reference\;area}$, area Stenosis $=1-\frac{\mathrm{lesion}\;\;area}{reference\;area}\;$. Intima-media area was calculated as the difference between the external elastic membrane (EEM) area and the lumen area. Radial artery diameter and luminal cross-sectional area were automatically measured using contour-detection software. Minimum lumen diameter and cross-sectional area at the site of dissection were assessed manually.

### 2.4 Data collection and statistical analysis

Demographic, clinical, procedural, catheter-related, and OCT-derived radial artery data were collected for all eligible patients. Clinical variables included age, sex, height, weight, body mass index, dyslipidemia, smoking status, hypertension, diabetes mellitus, renal insufficiency, cerebrovascular disease, peripheral artery disease, and prior transradial access.

Patients with OCT-detected RAD were compared with those without RAD in the full analyzable cohort. Categorical variables are shown as *n* (%) and were compared using the chi-square test or Fisher exact test, as appropriate. Continuous variables are shown as mean ± SD or median (IQR), depending on distribution, and were compared using the Student t-test or Wilcoxon rank-sum test, as appropriate. For paired baseline and repeat radial artery OCT measurements, paired t-tests or Wilcoxon signed-rank tests were used, as appropriate.

Exploratory logistic regression analyses were performed to identify factors associated with RAD occurrence. Variables were selected for the multivariable model on the basis of clinical relevance and univariable associations. Results are reported as odds ratios with 95% confidence intervals. A two-sided *P*-value < 0.05 was considered statistically significant. Data were organized and checked using Microsoft Excel (Microsoft Corp., Redmond, WA, USA). Statistical analyses were performed using Python version 3.11, and logistic regression models were fitted using the statsmodels package.

## 3 Results

Among the incidence cohort of 1,995 patients with analyzable radial OCT, RAD, with or without perforation, was identified in 266 patients (13.3%). Overall, 325 RAD lesions were identified, with a mean of 1.22 lesions per affected patient. Baseline, procedural, catheter-related, and OCT-derived radial artery characteristics according to RAD status are summarized in Table 1. In exploratory logistic regression analysis, older age, female sex, longer procedure duration, clinically relevant radial artery spasm, and smaller OCT-derived reference radial artery diameter remained associated with OCT-detected RAD (Table 2). A 5 Fr diagnostic catheter was used in 1,771 of 1,995 patients (88.8%), with similar use in patients with and without RAD (89.8% vs. 88.6%; *P* = 0.550). In the remaining patients, coronary angiography and PCI were completed using a single 6 Fr guiding catheter. PCI was performed using 6 Fr guiding catheters in both groups.

**Table 1:** Baseline and procedural characteristics according to the presence of radial artery dissection.

Variable	Overall (*N* = 1,995)	RAD (*n* = 266)	No RAD (*n* = 1,729)	*P*-value
Baseline clinical characteristics				
Age, years	60.11 ± 12.93	63.85 ± 13.31	59.53 ± 12.77	<0.001
Female sex, *n* (%)	411 (20.6)	84 (31.6)	327 (18.9)	<0.001
Body mass index, kg/m^2^	25.94 ± 3.45	25.40 ± 3.45	26.02 ± 3.45	0.007
Hypertension, *n* (%)	1,199 (60.1)	147 (55.3)	1,052 (60.8)	0.084
Diabetes mellitus, *n* (%)	625 (31.3)	84 (31.6)	541 (31.3)	0.925
Dyslipidemia, *n* (%)	973 (48.8)	130 (48.9)	843 (48.8)	0.972
Renal insufficiency, *n* (%)	103 (5.2)	16 (6.0)	87 (5.0)	0.500
Cerebrovascular disease, *n* (%)	214 (10.7)	30 (11.3)	184 (10.6)	0.755
Peripheral artery disease, *n* (%)	95 (4.8)	18 (6.8)	77 (4.5)	0.099
Current smoking, *n* (%)	1,294 (64.9)	156 (58.6)	1,138 (65.8)	0.023
Prior transradial access, *n* (%)	619 (31.0)	83 (31.2)	536 (31.0)	0.947
Prior statin therapy, *n* (%)	738 (37.0)	104 (39.1)	634 (36.7)	0.445
Prior aspirin therapy, *n* (%)	627 (31.4)	89 (33.5)	538 (31.1)	0.444
Prior P2Y12 inhibitor therapy, *n* (%)	436 (21.9)	59 (22.2)	377 (21.8)	0.890
Diagnosis, *n* (%)				0.302
STEMI	899 (45.1)	115 (43.2)	784 (45.3)	
NSTEMI	263 (13.2)	43 (16.2)	220 (12.7)	
UAP	833 (41.8)	108 (40.6)	725 (41.9)	
LDL-C, mmol/L	2.72 ± 0.98	2.74 ± 0.98	2.72 ± 0.98	0.759
Procedural and catheter characteristics				
Distal transradial access, *n* (%)	601 (30.1)	83 (31.2)	518 (30.0)	0.681
Anticoagulation category 2, *n* (%)	633 (31.7)	74 (27.8)	559 (32.3)	0.141
5 Fr diagnostic catheter used, *n* (%)	1,771 (88.8)	239 (89.8)	1,532 (88.6)	0.550
Number of 5 Fr diagnostic catheters	1 (1–1)	1 (1–1)	1 (1–1)	0.430
6 Fr guiding catheter used, *n* (%)	1,995 (100.0)	266 (100.0)	1,729 (100.0)	—
Number of 6 Fr guiding catheters	1 (1–1)	1 (1–1)	1 (1–1)	0.128
Total number of catheters	2 (2–2)	2 (2–2)	2 (2–2)	0.014
Clinically relevant radial artery spasm, *n* (%)	562 (28.2)	159 (59.8)	403 (23.3)	<0.001
Procedure duration, min	68 (56–84)	73 (59–91)	67 (55–84)	0.002
OCT-derived radial artery characteristics				
Reference radial artery diameter, mm	3.08 ± 0.28	3.01 ± 0.31	3.10 ± 0.28	<0.001

Values are presented as mean ± SD, median (IQR), or *n* (%). *P* values were calculated using the Student's t-test, Wilcoxon rank-sum test, chi-square test, or Fisher exact test, as appropriate. LDL-C, low-density lipoprotein cholesterol; NSTEMI, non-ST-segment elevation myocardial infarction; OCT, optical coherence tomography; RAD, radial artery dissection; STEMI, ST-segment elevation myocardial infarction; UAP, unstable angina pectoris.

**Table 2:** Exploratory logistic regression analysis for factors associated with radial artery dissection.

Variable	Univariable OR (95% CI)	*P*-value	Multivariable OR (95% CI)	*P*-value
Age, per 10 years	1.31 (1.18–1.46)	<0.001	1.17 (1.04–1.32)	0.008
Female sex	1.98 (1.49–2.63)	<0.001	1.80 (1.26–2.58)	0.001
Body mass index, per 1 kg/m^2^	0.95 (0.91–0.99)	0.007	0.96 (0.93–1.01)	0.085
Current smoking	0.74 (0.57–0.96)	0.023	0.97 (0.70–1.34)	0.854
Clinically relevant radial artery spasm	4.89 (3.74–6.40)	<0.001	4.97 (3.77–6.56)	<0.001
Procedure duration, per 10 min	1.06 (1.01–1.10)	0.015	1.08 (1.02–1.13)	0.003
Reference radial artery diameter, per 0.1-mm decrease	1.11 (1.06–1.16)	<0.001	1.11 (1.05–1.16)	<0.001

Odds ratios were derived from logistic regression models for OCT-detected RAD. The multivariable model included age, sex, body mass index, current smoking, procedure duration, clinically relevant radial artery spasm, and reference radial artery diameter. CI, confidence interval; OR, odds ratio; OCT, optical coherence tomography; RAD, radial artery dissection.

For all 325 RAD lesions, Type I–E1 was the most common subtype, accounting for 33.8% (110/325) of lesions. Detailed per-lesion morphometric and classification data are summarized in Supplementary Table S1 and Graphical abstract.

The repeat-OCT cohort included 22 patients (28 RADs) who underwent repeat radial OCT during subsequent TDRI procedures. Baseline characteristics of the repeat-OCT cohort are provided in Supplementary Table S2.

Among the 22 patients with 28 RADs identified by OCT, 5 patients (22.7%) underwent radial artery angiography during TDRI for technical difficulties: wire resistance in 1 case and catheter advancement resistance in 4 cases. Perforation was confirmed in 1 patient; the remaining 4 showed no angiographic evidence of dissection or perforation. Final angiography at procedure completion revealed no dissection or perforation in any patient (Supplementary Table S3).

In the repeat cohort, Type I–E1 comprised 35.7% (10/28). Hematoma was observed in five lesions (E1 = 2, E2 = 2, E3 = 1). Most dissections (96.4%) had an arc <90°, and nearly half occurred in sheath-unprotected segments. Detailed baseline OCT morphologic characteristics of the repeat-OCT RAD lesions, including dissection type, location, arc, and thrombus presence, are provided in Supplementary Table S4.

**Figure 3:**
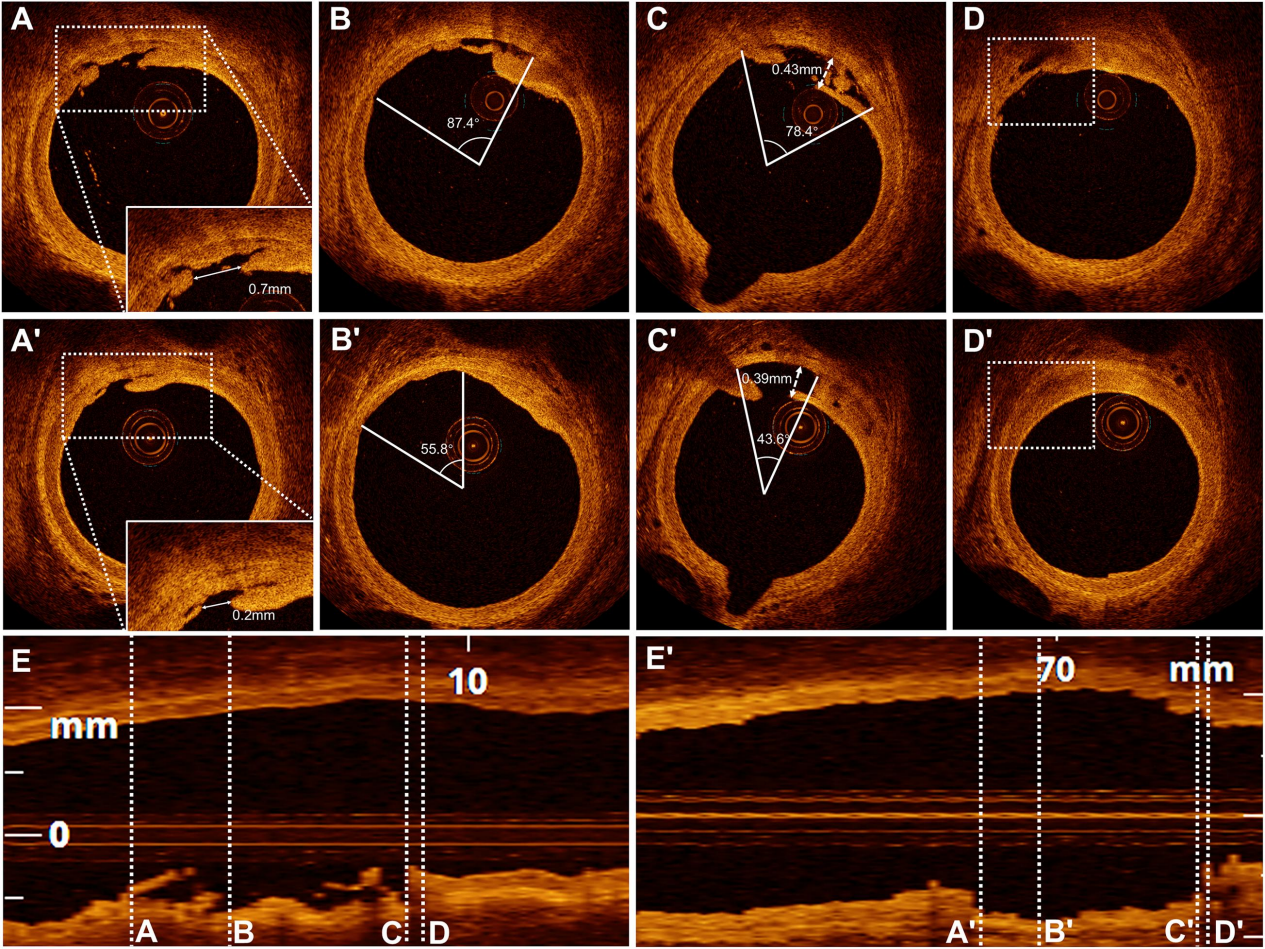
Representative case of radial artery dissection at baseline and 9-Day repeat radial artery OCT. **(A–D)** Baseline cross-sectional OCT images at the dissected segment: **(A)** cavity width (double arrow); **(B)** maximal dissection arc, 87.4°; **(C)** cavity depth, 0.43 mm, with a 78.4° arc; **(D)** dissection with associated hematoma.(A′–D′) Corresponding 9-day repeat radial artery OCT cross-sectional images at the same locations as **(A–D)**: (A′) cavity width decreased from 0.7 mm to 0.2 mm (white arrow); (B′) dissection arc decreased to 55.8°; (C′) cavity depth decreased to 0.39 mm; (D′) complete anatomic healing with restoration of wall integrity.(E and E′) Longitudinal OCT views of the dissected segment at baseline **(E)** and 9-day repeat-OCT (E′), with white dashed lines indicating the locations of cross-sectional images (A–D and A′–D′).

**Figure 4:**
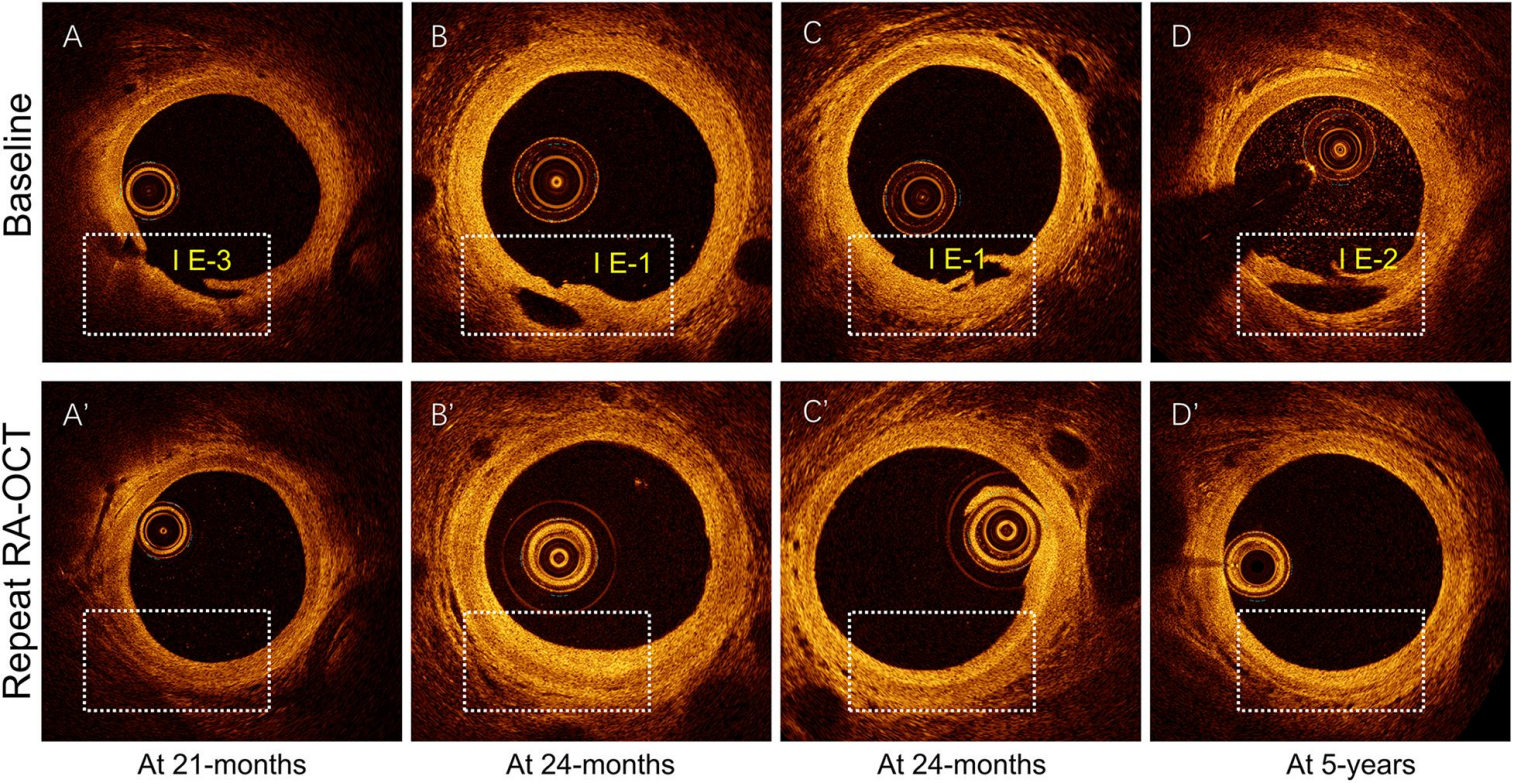
Representative cases of intimal hyperplasia healing pattern. **(A–D)** Baseline cross-sectional OCT images showing radial artery dissection subtypes. (A′–D′) Corresponding repeat-OCT images illustrating intimal hyperplasia with restoration of vessel wall architecture.

**Figure 5:**
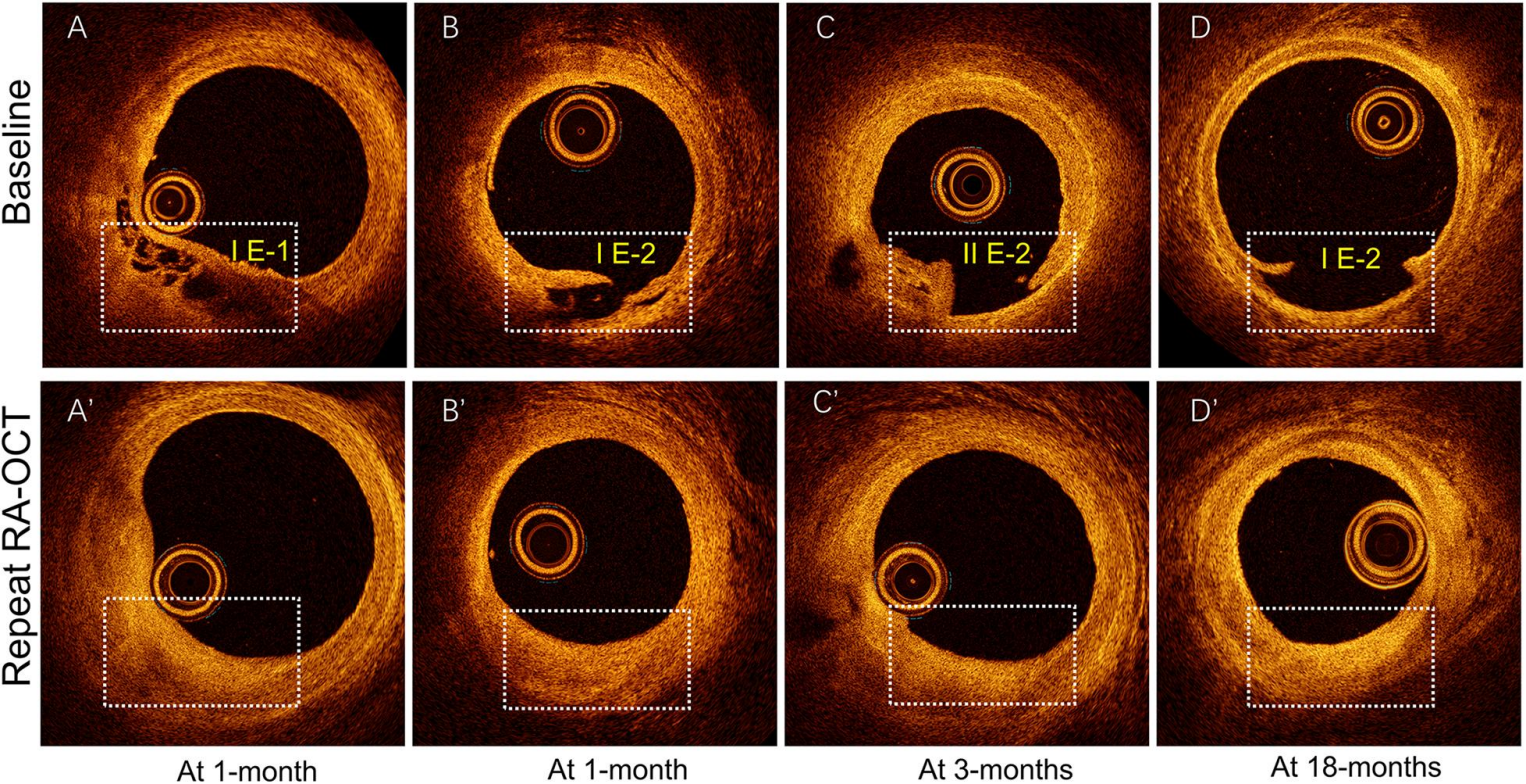
Representative cases of fibrous plaque healing pattern. **(A–D)** Baseline OCT images showing subtypes of radial artery dissection.(A′–D′) Corresponding repeat OCT images illustrating distinct healing patterns characterized by intimal hyperplasia.

**Figure 6:**
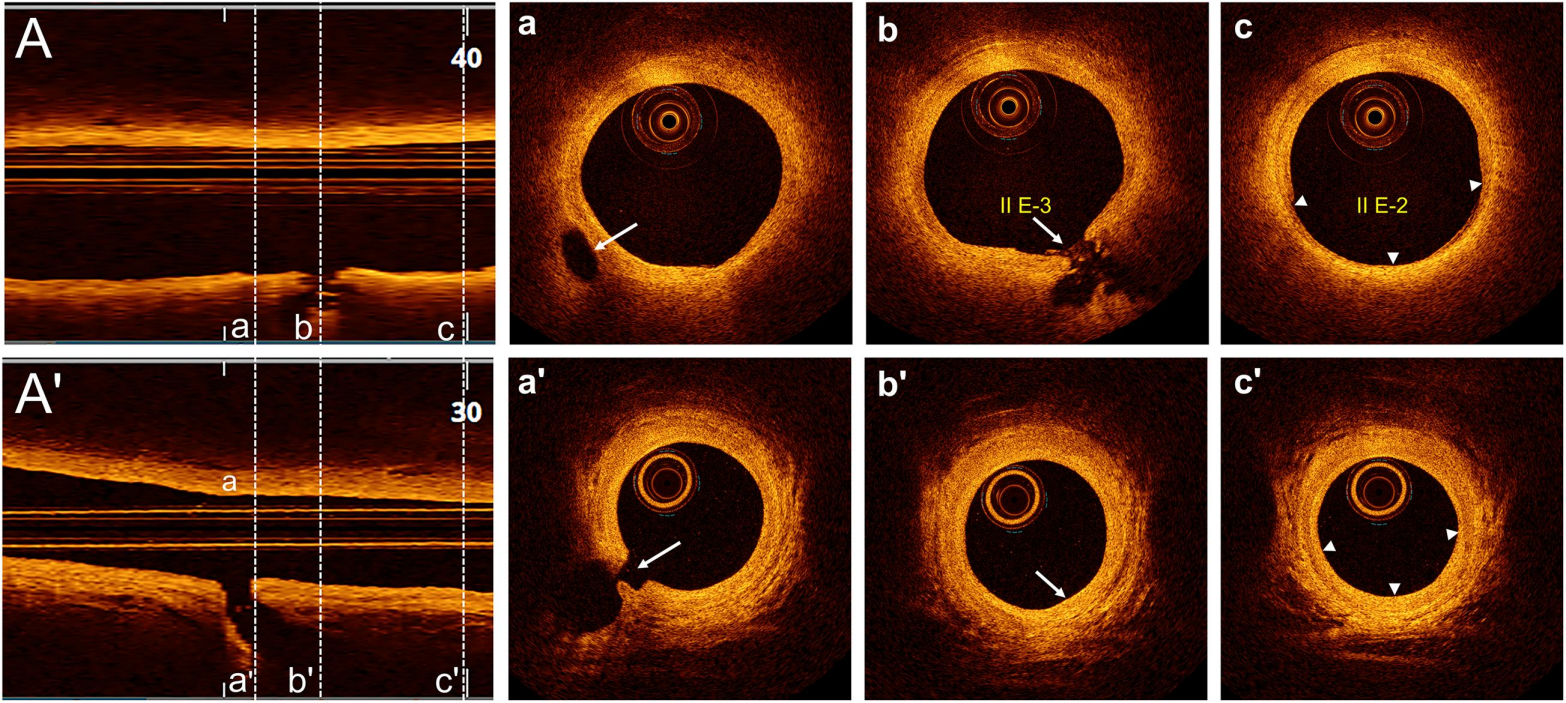
Healing response after radial artery perforation in a representative case. (A, A′) Longitudinal OCT views of the same radial artery segment at baseline and 5-year repeat-OCT.(a, a′) Bifurcation landmark (arrow).(b, b′) Perforation site at baseline (arrow in b) and corresponding intimal hyperplasia at 5-year repeat-OCT(b′).(c, c′) Dissection at baseline (arrowhead in c) with subsequent intimal hyperplasia at repeat-OCT (arrowhead in c′).

The median interval between OCT examinations was 153 days (IQR: 39.75–371). Repeat-OCT at day 9 in 1 patient with 3 RADs showed partial healing (Figure 3). The remaining 21 patients with 25 RAD lesions, who underwent repeat radial artery OCT ≥30 days after the index procedure, demonstrated complete healing at all dissection sites. Among these, 15 (60%) showed eccentric intimal hyperplasia and 10 (40%) had fibrous plaque formation (Figures 4–6)**.**

**Figure 7:**
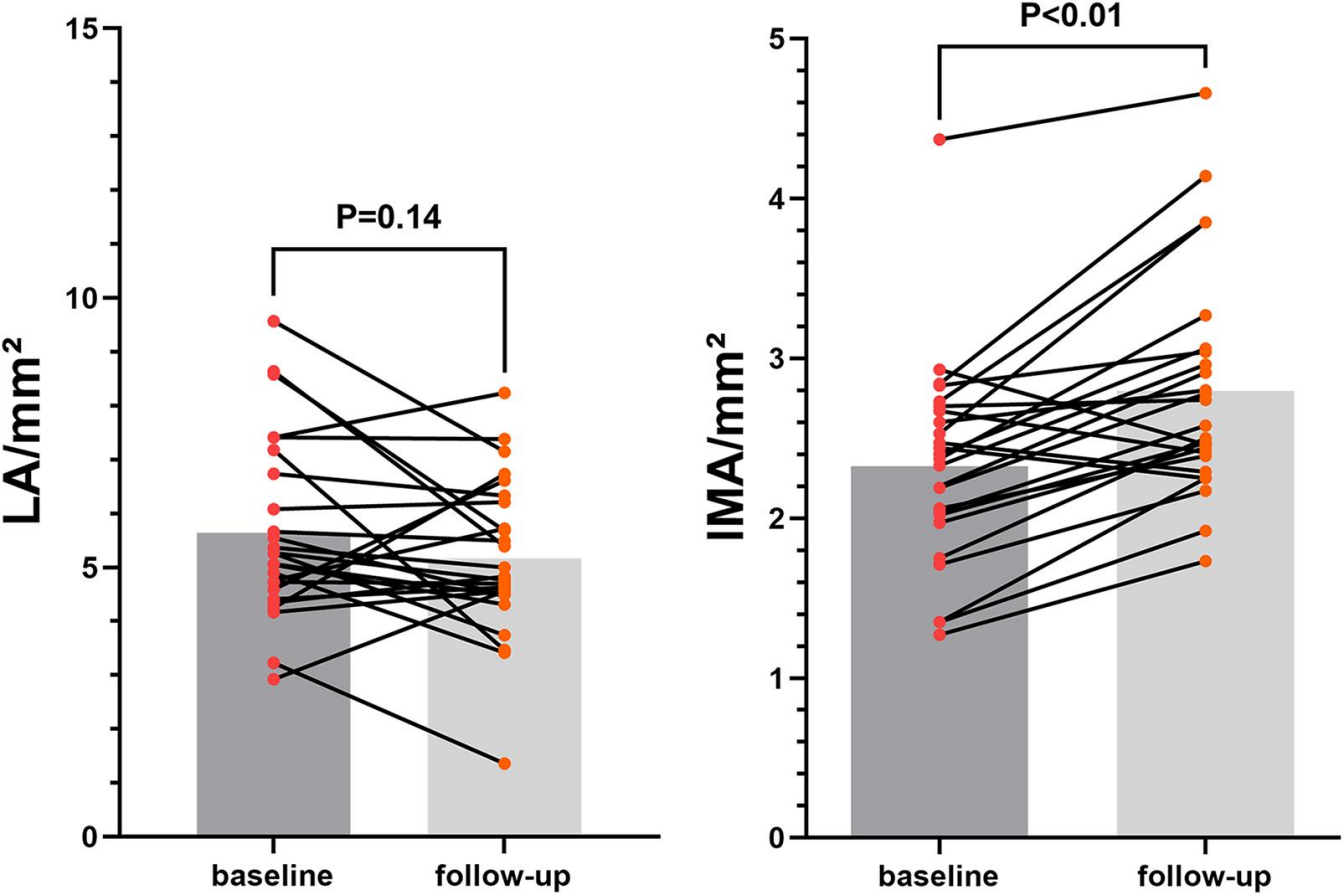
Quantitative changes in lumen area and intima-Media area after RAD healing. Paired OCT cross-sections at baseline and repeat-OCT showed preserved lumen area and a significant increase in intima–media area among 21 patients with 25 healed RAD of leisons (*P* < 0.01), indicating mild negative remodeling without appreciable luminal compromise. RAD, Radial artery dissection.

The mean remodeling index was 0.94 ± 0.11, corresponding to 12.5% mean area stenosis. The intima–media area increased significantly (2.32 ± 0.64 vs. 2.8 ± 0.75; *P* < 0.01), whereas the lumen area remained unchanged (5.65 ± 1.68 mm^2^ vs. 5.17 ± 1.47 mm^2^; *P* = 0.14). Figure 7 shows the longitudinal changes in lumen area and IMA; Table 3 summarizes baseline and repeat radial artery OCT measurements obtained during subsequent TDRI procedures in healed RAD lesions.

**Table 3:** Quantitative OCT measurements at baseline and repeat radial artery OCT in healed RAD lesions.

Variable	Baseline (*n* = 25)	Repeat radial artery OCT (*n* = 25)	*p*-Value (*n* = 25)
Lumen diameter, mm	2.65 ± 0.38	2.61 ± 0.55	0.74
Lumen area, mm^2^	5.65 ± 1.68	5.17 ± 1.47	0.14
Minimal lumen diameter, mm	2.52 ± 0.44	2.45 ± 0.39	0.48
Minimal lumen area, mm^2^	5.13 ± 1.77	4.85 ± 1.46	0.42
Reference vessel diameter, mm	2.78 ± 0.37	2.74 ± 0.32	0.6
Reference vessel area, mm^2^	6.17 ± 1.65	5.91 ± 1.41	0.42
Intima–media area, mm^2^	2.32 ± 0.64	2.8 ± 0.75	<0.01
External elastic membrane area, mm^2^	8.0 ± 1.96	8.07 ± 1.93	0.85

Values are presented as mean ± SD.

## 4 Discussion

The principal findings of this study were: (1) In 1,995 patients undergoing 6F TDRI, RAD occurred in 13.3%; 325 RAD lesions were identified (mean 1.22 per affected patient), with Type I–E1 predominant (33.8%). (2) At ≥30-day repeat radial artery OCT (21 patients; 25 lesions), all dissections healed completely, mainly via intimal hyperplasia or fibrous plaque with a mean remodeling index of 0.94 ± 0.11 (mild negative remodeling).

### 4.1 Incidence of RAD

The reported incidence of RAD following TDRI varies considerably depending on the imaging modality used. Angiographic studies have reported an incidence ranging from 0.6% to 10% (5, 21), whereas ultrasound has detected RAD in approximately 1.2% of cases (6), In contrast, owing to its superior axial resolution, has consistently demonstrated higher detection rates, with previously reported incidences ranging from 16.5% to 35.6% (8, 9). In the present study, which represents the largest sample based on OCT to date, the incidence of RADs detected by OCT was 13.3%, consistent with previous OCT-based findings. This variability likely reflects not only the inherent differences in imaging resolution but also methodological factors, including retrospective study designs, limited sample sizes, and heterogeneity in sheath type and diameter.

### 4.2 Classification of RAD

OCT imaging revealed a wide morphological spectrum of RADs following TDRI. To facilitate structured analysis, we proposed an OCT-based classification system incorporating both geometric pattern and injury depth, adapted from established SED classification (13, 15). Dissections were categorized as Type I (flap) or Type II (cavity) based on their appearance. Each type was further stratified into subtypes E1–E3, yielding six distinct categories: Type I-E1 to I-E3 and Type II-E1 to II-E3.

Overall cohort (325 RAD lesions): Type I–E1 was the most frequent subtype (33.8%). Type I–E1 plus Type I–E2 together accounted for 59.7%, indicating that most injuries were shallow (within/at the EEM) rather than transmural. In contrast, flap-type injuries predominate in coronary stent-edge dissections (>95%) (13, 15). Repeat-OCT cohort (28 lesions): Type I accounted for 35.7% and Type II–E2 for 21.4%, a distribution consistent with the overall cohort.

This subclassification reflects a gradation of structural damage to the arterial wall, which may correlate with healing responses. However, the small sample size and wide variability in follow-up intervals (9 days to 5 years) limit the ability to draw definitive conclusions regarding the relationship between dissection subtype and healing pattern. Nonetheless, this classification may serve as a useful framework for assessing the morphology and severity of RAD and can guide future research into its prognostic significance.

### 4.3 Potential mechanisms and clinical implications of RAD

RAD is more likely to occur in anatomically challenging settings, such as radial loops, tortuous segments, arterial spasm, or small-caliber vessels (22, 23). Mechanistically, RAD may result from direct mechanical injury during guidewire, sheath, or catheter advancement, including the catheter-edge “razor effect,” inadvertent guidewire entry into small side branches, and vessel–sheath mismatch with secondary vasospasm and friction (23–25).

In the full analyzable cohort, older age, female sex, longer procedure duration, clinically relevant radial artery spasm, and smaller OCT-derived reference radial artery diameter were associated with OCT-detected RAD. These findings are consistent with prior studies showing that patient-related factors, vessel caliber, radial artery spasm, and procedural complexity are associated with radial access complications (2, 22, 26, 27). Among these factors, radial artery spasm and smaller radial artery diameter are particularly relevant because they reduce the functional lumen and may amplify vessel–device mismatch and friction during a uniform 6 Fr sheath-based procedure (2, 26). Catheter-size effects could not be assessed because PCI was performed using 6 Fr guiding catheters in both groups, whereas 5 Fr catheters were diagnostic catheters.

Clinically, prevention should focus on reducing radial artery spasm and avoiding forceful guidewire, sheath, or catheter manipulation. Adequate vasodilator use, gentle device advancement, early recognition of resistance, and access-site conversion when necessary may reduce arterial injury (2, 22). Within the repeat-OCT cohort, uncomplicated OCT-detected RAD lesions showed a favorable healing pattern, with complete healing of all lesions reassessed ≥30 days and only mild negative remodeling.

### 4.4 Healing time and healing pattern

Previous studies have shown that endothelium-dependent flow-mediated dilation of the radial artery typically recovers within approximately three months following TDRI (28), However, data on the structural repair timeline of RAD remain scarce. OCT assessments of SEDs have reported variable healing rates: incomplete healing was observed in 8.3%–8.6% of cases at 6 months, in 28.5% at 9 months, and in 10% at 12 months (15). These discrepancies likely reflect differences in patient characteristics, stent platforms, and follow-up durations.

In contrast, our study demonstrated a distinctly faster healing course for RAD. Incomplete healing was observed in only one patient (3 RADs) with a 9-day repeat-OCT, whereas the remaining 21 patients (25 RADs) with follow-up ≥30 days showed complete dissection resolution. These data suggest that RAD may repair more rapidly than coronary SED, although this comparison should be interpreted cautiously because of differences in vessel type, injury mechanism, and study design (9, 10). Repeat-OCT confirmation of structural healing may provide more detailed information than macroscopic patency alone when considering future reuse of the radial artery. This approach may help refine decisions about radial artery reuse in selected patients.

Regarding healing patterns, among the 21 healed RADs, 60% exhibited an intimal proliferation pattern, while 40% showed fibrous plaque formation. Prospective studies with larger sample sizes are warranted to further investigate the determinants of RAD healing pattern.

### 4.5 Radial artery remodeling following dissection

Previous studies of transradial intervention have reported pronounced inward remodeling of the radial artery, characterized by increased intimal volume, elevated intima-to-media area ratio, greater maximal intimal thickness, and resultant luminal narrowing. These changes are primarily attributed to neointimal hyperplasia, driven by smooth muscle cell proliferation, migration, and extracellular matrix deposition (8, 29). In our cohort, the mean remodeling index was mildly reduced (<1.0), accompanied by a slight increase in the intima-to-media area ratio and a modest reduction in mean lumen area. Collectively, these findings support the concept that post-dissection remodeling reflects a compensatory, and predominantly benign, vascular response, consistent with previous OCT-based observations.

### 4.6 Mechanisms of RAD healing

The healing of RADs after catheterization shares the “layering” or “tacking down” mechanism described in SEDs (15, 30), but differ from SEDs by exhibiting pronounced intima-media thickening despite a unchanged lumen, reflecting vessel-specific repair processes. Several mechanisms may account for the more rapid and structurally distinct healing observed in RADs: 1) Vascular composition: The radial artery has a higher density of smooth muscle cells compared with coronary arteries, favoring a proliferative wound-healing response dominated by smooth muscle hyperplasia (31, 32). 2) Plaque burden at the dissection site: RADs typically occur in relatively plaque-free segments, whereas SEDs originate in regions that are variably affected by atherosclerosis (15, 33). 3) Hemodynamic forces: Radial arteries are exposed to sustained, laminar flow, which generates physiologic shear stress that promotes endothelial regeneration and adaptive vascular remodeling (34–36). In contrast, disturbed, low-shear flow in stenotic or irregular coronary segments impairs endothelial repair and delays healing (37). 4) Flow directionality: The retrograde propagation pattern of many RADs may facilitate flap apposition and healing, a phenomenon similarly observed in spontaneous coronary artery dissections, where retrograde flow has been linked to improved outcomes (38). 5) Mechanical tamponade: Catheter-induced radial wall compression may exert a tamponade effect on minor dissections or perforations, often leading to spontaneous sealing and obviating the need for additional intervention (22). Collectively, these five mechanisms not only support the accelerated and consistent healing observed in RADs but also explain the predominance of intimal fibrous hyperplasia (60%) and neointimal plaque formation (40%), reflecting a proliferation-driven reparative response unique to the radial artery.

### 4.7 Study limitations

This study has several limitations. First, the single-center, retrospective design may limit the generalizability of the findings. Although baseline, procedural, catheter-related, and OCT-derived radial artery characteristics were analyzed in the full analyzable cohort, the regression analysis was exploratory; residual confounding cannot be excluded, and these findings should therefore be interpreted as hypothesis-generating. Second, the absence of a standardized post-procedural repeat imaging interval resulted in variable imaging intervals, which hindered precise characterization of the temporal course of RADs healing. Third, the study was underpowered to evaluate the clinical significance of OCT-detected RADs and to compare differences among distinct repair patterns. Fourth, owing to the retrospective nature of the study, systematic postoperative ultrasound imaging was not available for all patients. Consequently, the influence of RADs on the true incidence of RAO could not be comprehensively assessed. Fifth, all procedures were performed using a 6 Fr, 16-cm Radiofocus Introducer II sheath (Terumo Corp., Tokyo, Japan), and PCI was performed using 6 Fr guiding catheters. Therefore, the findings should be interpreted within the context of a uniform 6 Fr sheath-based and 6 Fr guiding catheter strategy and may not be generalizable to other sheath sizes, sheath types, sheathless techniques, or different guiding catheter sizes. Future prospective studies with larger cohorts and predefined, uniform follow-up protocols are needed to validate the factors associated with RAD and to elucidate the mechanistic determinants, temporal evolution, and clinical relevance of RADs healing**.**

## 5 Conclusion

In this 6 Fr sheath-based TDRI cohort, in which PCI was performed using 6 Fr guiding catheters, RAD was frequently detected by OCT, occurring in 13.3% of patients. Type I–E1 was the predominant subtype in the full RAD lesion cohort, consistent with limited medial injury without EEM disruption. In the repeat-OCT cohort, lesions assessed ≥30 days showed complete healing, mainly through intimal hyperplasia or fibrous plaque formation, with mild negative remodeling and preserved luminal caliber. These findings suggest a favorable healing pattern for uncomplicated OCT-detected RAD, although its long-term clinical relevance requires further study.

## Data Availability

The raw data supporting the conclusions of this article will be made available by the authors, without undue reservation.
