# Supplementary material for: Incidence, classification, healing patterns, and vascular remodeling of radial artery dissection assessed by OCT
Source: Front Cardiovasc Med. 2026 Jun 3;13:1754057. doi: 10.3389/fcvm.2026.1754057 (PMC13273964; doi:10.3389/fcvm.2026.1754057)
Supplement: Supplementary file 1 [file Datasheet1.docx]

**Supplementary Table S1. Characteristics of RAD on OCT (N=325)**

| Variables | Total (n = 325) |
| --- | --- |
| Flap |  |
| Type I-E1 | 110(33.8) |
| Type I-E2 | 84(25.8) |
| Type I-E3 | 41(12.6) |
| Cavity |  |
| Type II-E1 | 34(10.5) |
| Type II-E2 | 32(9.8) |
| Type II-E3 | 24(7.4) |
| RAD measurements |  |
| Distance from ostium of RA to RAD site (mm) | 64.5(27.25,140) |
| RAD length (mm) | 4.2(1.8, 8.2) |
| Maximum RAD Arc (°) | 42.8±21.8 |
| Small dissection (Arc＜90°) | 296(91.1) |
| Maximum length of flap (mm) | 0.59±0.23 |
| Maximum thickness of flap (mm) | 0.17±0.06 |
| Maximum area of dissection (mm^2^) | 0.2 (0.11, 0.41) |
| Maximum width of dissection (mm) | 1.4±0.69 |
| Maximum depth of dissection (mm) | 0.22±0.09 |
| Values are n (%), mean ± SD, or median (interquartile range). Abbreviation: RAD = radial artery dissection; OCT=optical coherence tomography | |

**Supplementary Table S2. Baseline characteristics of the repeat-OCT cohort**

| Variable | Value  (n=22) |
| --- | --- |
| Age, y | 58.9 ± 13.8 |
| Male ,n(%) | 18 (81.8) |
| Body mass index, kg/m² | 24.5 ± 1.9 |
| Previous PCI or angiography, n(%) | |
| Radial artery access | 5 (22.7) |
| Distal radial artery access | 1 (4.5) |
| Diagnosis, n(%) | |
| STEMI | 12 (54.5) |
| NSTEMI | 1 (4.5) |
| UAP | 9 (40.9) |
| Comorbidities, n(%) | |
| Hypertension | 12 (54.5) |
| Hyperlipidemia | 7 (31.8) |
| Diabetes | 6 (27.3) |
| Peripheral vascular diseases | 2 (9.1) |
| Ischemic cerebrovascular disease | 4 (18.2) |
| Medications, n(%) | |
| Aspirin | 6 (27.3) |
| ACEI/ARB | 4 (18.2) |
| Beta-blockers | 4 (18.2) |
| Calcium channel blockers | 4 (18.2) |
| Statins | 5 (22.7) |
| Laboratory findings | |
| Triglycerides, mmol/L | 1.24(0.97,1.69) |
| Glucose, mmol/L | 6.88(4.99,10.05) |
| LDL-C, mmol/L | 2.75 ± 1.04 |
| Time between baseline and follow-up (d) | 153.5 (39.75, 371) |
| Values are n (%), mean ± SD, or median (25th,75thpercentiles). Abbreviations: ACEI = angiotensin converting enzyme inhibitor; ARB = angiotensin receptor blocker; BMI = body mass index; LDL-C = low-density lipoprotein cholesterol; NSTEMI = non–ST-elevation myocardial infarction; PCI = percutaneous coronary intervention; STEMI = ST-elevation myocardial infarction; UAP = unstable angina pectoris | |

**Supplementary Table S3. Procedural Characteristics Of the repeat-OCT cohort**

| Variable | Value  (n=22) |
| --- | --- |
| Initial intervention access, n(%) | |
| Radial access | 8 (36.4) |
| Distal radial access | 14 (63.6) |
| Follow-up intervention access, n(%) | |
| Radial access | 8 (36.4) |
| Distal radial access | 14 (63.6) |
| Sheath size 6Fr, n(%) | 22 (100) |
| Pre-procedure RAA, n(%) | 5 (22.7) |
| Dissection/perforation, n(%) | 1(4.5) |
| Anticoagulation, n(%) | |
| Heparin | 17 (77.3) |
| Bivalirudin | 5 (22.7) |
| 6F guiding catheter | 22 (100) |
| Target vessel, n(%) | |
| LAD | 14 (63.6) |
| LCX | 1 (4.5) |
| RCA | 7 (31.8) |
| Post-procedure RAA, n(%) | 22 (100) |
| Dissection/perforation, n(%) | 0 (0) |

Values are n (%), mean ± SD, or median(25th,75thpercentiles). Abbreviations: LAD = left anterior descending artery; LCX = left circumflex artery; RCA = right coronary artery; RAA = radial artery angiography.

**Supplementary Table S4. Detailed baseline OCT morphologic characteristics of the repeat-OCT RAD lesions**

| Variable | Value  (n=22) |
| --- | --- |
| Flap, n(%) | |
| Type I-E1 | 10 (35.7) |
| Type I-E2 | 6 (21.4) |
| Type I-E3 | 3 (10.7) |
| Cavity, n(%) | |
| Type II-E1 | 4 (14.3) |
| Type II-E2 | 3 (10.7) |
| Type II-E3 | 2 (7.1) |
| Distance from ostium of RA to RAD site (mm) | 98.1 ± 60.3 |
| Length of analyzed RA (mm) | 203.5 ± 17.7 |
| RAD measurements | |
| RAD length (mm) | 3.8 (1.9, 9.3) |
| Maximum RAD Arc (°) | 45.3 ± 21.8 |
| Small dissection (Arc <90°) | 27 (96.4) |
| Maximum length of flap (mm) | 0.63 ± 0.38 |
| Maximum thickness of flap (mm) | 0.17 ± 0.07 |
| Maximum area of dissection (mm²) | 0.23 (0.13, 0.4) |
| Maximum width of dissection (mm) | 0.93 ± 0.81 |
| Maximum depth of dissection (mm) | 0.27 ±0.14 |
| White thrombus, n(%) | 16 (57.1) |
| RAD in sheath-unprotected segment, n(%) | 13 (46.4) |

Values are n (%), mean ± SD, or median (25th,75thpercentiles). Abbreviation: RAD = radial artery dissection; OCT=optical coherence tomography

**Supplementary Video S1.** Off-wire radial artery OCT pullback with co-registration.

The video shows the OCT catheter position together with cross-sectional and longitudinal OCT images during off-wire radial artery pullback.
